# Dietary Supplements and the Skin: Focus on Photoprotection and Antioxidant Activity—A Review

**DOI:** 10.3390/nu14061248

**Published:** 2022-03-16

**Authors:** Thalita Marcílio Cândido, Maíra Bueno Ariede, Fabiana Vieira Lima, Luciana de Souza Guedes, Maria Valéria Robles Velasco, André Rolim Baby, Catarina Rosado

**Affiliations:** 1 Department of Pharmacy, Faculty of Pharmaceutical Sciences, University of São Paulo, São Paulo 05508-900, Brazil; thalitamcandido@gmail.com (T.M.C.); maariede@gmail.com (M.B.A.); fabianavlimag@gmail.com (F.V.L.); lsouguedes@hotmail.com (L.d.S.G.); mvrobles@usp.br (M.V.R.V.); 2 CBIOS, Universidade Lusófona’s Research Center for Biosciences & Health Technologies, 1749-024 Lisbon, Portugal

**Keywords:** food supplement, skin health, photoprotection

## Abstract

Skin health is not only significantly affected by ageing, but also by other lifestyle-related factors, such as sun exposure, exercise and eating habits, smoking or alcohol intake. It is known that the cutaneous tissue can exhibit visible signs of senescence, in the form of, for example, dull complexion, loss of firmness, or changes in pigmentation. Consumers attempt to improve skin health and appearance not only by cosmetic products, but also with the consumption of food supplements. Recently, there has been an increase in the amount of food supplements with claims that are related to skin and hair health. Nevertheless, the literature is still scarce in evidence of the efficacy of this type of products. Considering this scenario, we aim in this review to assemble studies and methodologies that are directed at the substantiation of the cutaneous health claims of food supplements. For example, we reviewed those that were indicative of antioxidant properties, improvement in pigmentation disorders, increased hydration or protection against the damages caused by ultraviolet radiation.

## 1. Introduction

The food supplement market has an extensive range of products with the most diverse health benefit claims. Among these benefits, a few are aimed at maintaining skin health and improving its appearance, but often scarce scientific studies are found to support such claims. However, several methodologies applicable to in vitro and in vivo studies are currently available to demonstrate the beneficial effects of the substances contained in these products on the skin, such as hydration improvement, protection against ultraviolet (UV) radiation or the reduction in wrinkles and spots. As examples of methodologies, we can mention the measurement of gross and net elasticity of the skin using the equipment Cutometer^®^, the evaluation of erythema and melanin with the use of a Mexameter^®^, the evaluation of wrinkle depth using a three-dimensional microtopography imaging system (PRIMOS 3D lite) or the Visia^®^ equipment, among others, which will be mentioned in this review [1,2,3,4,5,6,7,8,9].

Exposure to solar radiation is one of the main factors that contribute to skin aging, since ultraviolet (UV) radiation stimulates the formation of free radicals, which can cause oxidative stress. Oxidative stress in the skin is related to mutations and photoaging. Photoaging is an accumulative process and dependents on the rate and frequency of sun exposure, as well as skin phototype (skin pigmentation). UVA radiation can penetrate through the epidermis to the dermis, causing damage to the connective tissue, or extracellular matrix (ECM), represented here by collagen, elastin and glycosaminoglycanes (GAGs). Damage to these structures generates visible damage in the skin, such as loss of elasticity, hydration and skin firmness, resulting in the appearance of wrinkles and increased fragility, for example. UVB radiation, on the other hand, is absorbed in the epidermis by DNA, RNA and protein aromatic amino acid molecules, among other molecules, causing direct DNA damage, erythema, stratum corneum thickening, melanogenesis and photoimmunosuppression. The absorption of UVB radiation by DNA and RNA bases can lead to various mutations and affect cellular protein synthesis. The accumulations of unrepaired mutations can cause cell cycle arrest and apoptosis. Furthermore, mutations can nullify the apoptotic capacity of cells and thus increase the formation of malignancies. Such a result will depend on the skin cell type, UV wavelengths and cumulative UV dose [10,11,12,13].

In this review, we present studies that established efficacy evidence and support skin claims for some of the most common bioactives used in dietary supplements. Although there are several other food supplements that have a positive influence on skin health conditions, such as Vitamins C and E, Coenzyme Q10 and probiotics [14], the authors chose rosmarinic acid, *Polypodium leucotomus*, Pycnogenol^®^, astaxanthin, lutein, tranexamic acid, pomegranate (*Punica granatum* L.) extract and orthosilic acid since these supplements are potential trends in the market and studies demonstrate their efficacy in the improvement of the aspect and healthy of the skin. For each compound or substance, a specific introduction is provided, highlighting the mechanism that may be associated with their skin bioactivity, followed by the results obtained in relevant in vitro and in vivo investigations. Another objective of this review is to provide useful material for researchers aiming to further establish, particularly in vivo, the skin beneficial effects of dietary supplements.

## 2. Rosmarinic Acid

**Figure 1:**
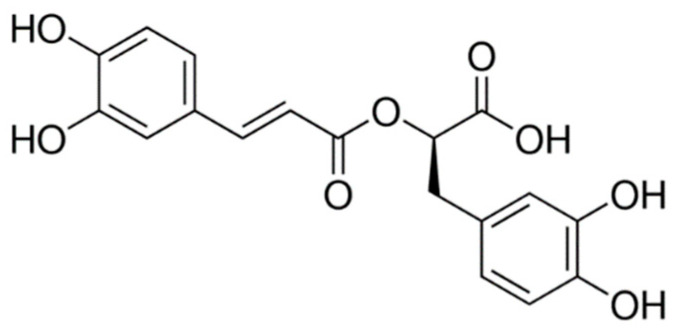
Rosmarinic acid molecular structure (C_18_H_16_O_8_).

Rosmarinic acid, or (R)-1-carboxy-2-(3,4-dihydroxyphenyl) ethyl ester of 3,4-dihydroxy cinnamic acid, is an ester of caffeic acid and 3,4-dihydroxyphenylacetic acid, being found in plants, such as rosemary (*Rosmarinus officinalis*). The molecular structure of the molecule is represented in Figure 1 [15,16,17].

Rosmarinic acid molecule has interesting biological activities, such as antiviral, antibiotic, anti-inflammatory, immunomodulatory (tested in the treatment of atopical dermatitis), anticarcinogenic and antioxidant properties [18]. Recent studies showed that rosmarinic acid can reduce proinflammatory lysophosphatidylcholine production, hinder vitamin E depletion and inhibit the oxidation of low-density lipoproteins (LDL). Fernando et al. [16] demonstrated the cytoprotective effect of rosmarinic acid against oxidative stress induced by ultraviolet B (UVB) radiation. HaCaT (Cultured Human Keratinocyte) cells treated with rosmarinic acid prior to UVB irradiation showed an increase in the activity of the antioxidant system and a lesser oxidative damage to the biological macromolecules, observed through intracellular ROS detection, lipid peroxidation and DNA fragmentation assays, for example in [19].

It is known that rosemary has a high content of rosmarinic acid. For this reason, several laboratories have developed rosemary supplements claiming benefits, such as skin quality improvement and skin photoprotection, which are based on the antioxidant property of the rosmarinic acid to effectively prevent damage caused by UV radiation [2,7,16,20,21,22].

Pérez-Sánchez et al. [21] studied the protective activity of the NutroxSun^®^ product, containing extracts of *Rosmarinus officinalis* and grapefruit (*Citrus paradisii*) on HaCaT keratinocytes. To evaluate the effect of RA on HaCaT cells in ROS generation, for example, 2′,7′-dichlorodihydrofluorescein diacetate (H_2_DCFDA) was used to monitor intracellular UVB-induced ROS generation. For this purpose, the cells were treated with a thin layer of PBS containing the RA extract (75–100 lg/mL), followed by exposure to UVB light (800 or 1200 J/m^2^) and labeled with H_2_DCFDA (fluorescent probe that becomes fluorescent when oxidized by free radicals). The treatment with RA extract was able to decrease ROS formation. Additionally, its photoprotective activity in volunteers was assessed by the determination of the minimal erythema dose (MED) after oral ingestion at different time points. The skin protective effect against UV radiation was determined by the calculation of the MED before treatment and after 29, 57 and 85 consecutive days of ingestion of the food supplement (250 mg, one capsule per day). The MED was calculated inducing erythema by an artificial light source with a filtering system (Multiport^®^ Solar Simulator; Solar Light Co., Glenside, PA, USA). An increase of 37% was observed in the MED (*p* < 0.05) after 8 weeks of treatment and an even greater MED was obtained after 12 weeks (56%, *p* < 0.01).

In a similar fashion, Nobile et al. [7] investigated the effect of rosemary and grapefruit extracts on skin photoprotection through a MED assay. For the assessment of the minimal erythemal dose, series of UVB doses were applied on small subsites of the skin of the back of the volunteers. The skin redness caused by UVB radiation was measured by spectrophotometer/colorimeter CM-700D (Konica Minolta, Milano, Italy) in the CIELab color space (a*, red-green parameter). Volunteers were divided in three groups—untreated control, administration of 100 mg of capsules containing the extracts, and administration of 250 mg of the same sample. The results showed in both dose groups, a significant rise of the MED. In the 100 mg dose group, MED increased 15.2%, 20.5%, and 29.8% mJ/cm^2^, after 0.5, 1, and 2 months of treatment, respectively. A similar efficacy was noticed in the other dose group. Although the variation of MED was not statistically significant when 100 and 250 mg data were compared, a significance was reached when these were compared with the placebo group. The authors also verified other skin parameters, such as lipid peroxidation, elasticity and wrinkle depth. The latter was measured in the periocular area using a three-dimensional microtopography imaging system (PRIMOS 3D lite, GFMesstechnik GmbH, Teltow, Germany). A noteworthy decrease in wrinkle depth was observed for both dose groups when compared to the control, but, again, the variation of wrinkle depth was not statistically significant when the two-dosage data were compared. For the skin elasticity assessment, the researchers studied the gross and net elasticity, using the Cutometer^®^ MPA 580 (Courage + Khazaka Electronic, Köln, Germany). The skin surface of the face cheeks of the volunteers was analyzed. For this purpose, the skin surface of volunteers’ cheeks was drawn into the probe by a negative pressure for 3 s and released for 3 s. The penetration depth of the skin inside the probe, during the suction and the release phase, was measured by an optical system. The same variation of skin elasticity, regarding to gross elasticity, was observed when the two dosages were compared. The results of the lower-dose group indicated an increase in the skin elasticity, 1.8, 3.2, and 4.6%, after 0.5, 1, and 2 months of treatment, respectively, whereas in the 250 mg dose group, it increased by 1.5, 2.9, and 3.7%. Towards skin net elasticity, a significant increase was also observed for both dosage groups. For the assessment of basal and UVA-stimulated (10 J/cm^2^) lipid peroxidation (LPO), a tape-stripping technique was employed to determine the concentration of malondialdehyde (MDA) using Corneofix^®^ foils (Courage + Khazaka Electronic, Köln, Germany) to obtain stratum corneum samples from the back of volunteers. The stratum corneum MDA content after 4 h of UVA exposure decreased by 9.7, 16.2 and 20.1%, and a similar decrease was observed after 24 h.

In summary, skin improvements were observed in the volunteers that received the treatment, although no statistic differences were seen between the 100 and 250 mg dose treatment, for the parameters investigated. The presumptive mechanism for the differences between the control and the other two groups has been attributed to rosmarinic acid and its antioxidant properties, causing the inhibition of reactive oxygen species (ROS) induced by UV radiation and concomitant inflammatory markers (LPO and cytokines) combined with a blockage of intracellular signaling pathways, which cause extracellular matrix degradation [2,16,23,24,25,26].

## 3. *Polypodium leucotomus*

*Polypodium leucotomus* (PL) is a tropical fern species in the Polypodiaceae family [27,28]. Its aqueous extract is obtained from the leaves and known commercially as Fernblock^®^. It is used as an oral and topical photoprotector agent [29]. Phytochemical studies showed a high concentration of polyphenols, monosaccharides (mainly fructose and glucose), quinic, shikimic, glucuronic, and malic acids [30,31]. The high antioxidant activity and photoprotective properties were attributed to the high content of phenolic components: p-coumaric, caffeic, ferulic, and chlorogenic acids [32,33,34]. Several studies have been conducted to unveil the mechanisms of PL photoprotective effect, mainly through in vitro or in vivo (animal model) studies [29,31].

Various investigations attributed the PL mechanism of action to an antioxidant action inhibiting ROS formation, especially those induced by UV [34,35], as well as an immunomodulatory pathway and anti-inflammatory effect [27,32,36,37,38].

Both the caffeic and ferulic acids presented in PL extract are antioxidants, inhibit UV-mediated peroxidation and are effective in protecting the skin from UVB-induced erythema [31,39]. The caffeic acid has anti-inflammatory properties by 5-lipoxygenase inhibition followed by leukotriene biosynthesis [40]. Furthermore, PL inhibited the photoisomerization of trans-urocanic acid, a chromophore found in the skin, which after UV-induced isomerization has immunosuppressive activity [41]. However, the exact mechanism is not clear, and could be based on the antioxidant effect of PL, but the literature does not attribute ownership to a specific group or component of the extract.

A PL beneficial effect was found on the regulation of matrix metalloproteinase that transformed growth factor-B and fibrillar collagen using a culture of dermal fibroblasts, melanoma cells and UV-radiated fibroblasts. The authors attributed the effect to the TGF-β expression on cell-specific regulation and to the antioxidant effect on the fibroblasts and melanoma cells [42].

Regarding toxicological data by Murbach et al. [43], no evidence of toxicity, genoxicity, and mutagenicity was found in a repeated oral dose animal study.

Kohli et al. [44] investigated the molecular and photobiological effects of oral PL administration. Subjects were irradiated with visible light, UVAI, and UVB for 4 days, and on days 3 and 4, the evaluation occurred after ingestion of PL. Clinical and colorimetric trials showed a decrease in erythema after UVB-induced irradiation in 17 out of the 22 subjects after PL oral administration. UV damage biomarkers related to apoptosis and DNA injury were found to be decreased in all subjects through the histological findings. These results seemed to suggest that PL could be used as an adjuvant against UVB photobiological effects.

Oral PL was investigated as a complement in the treatment of vitiligo and presented relevant results in repigmentation improvement. However, no studies assessed its effectiveness as the only form of treatment [35,45]. Some clinical studies investigated PL as a treatment of melasma using the melasma severity index evaluation, showing promising results [45]. However, Ammar investigated oral PL and compared it with a placebo, not obtaining differences between the two groups by narrowband reflectance spectrophotometry, nor by the severity index [46].

To date, no specialized studies have been published to confirm the post-inflammatory hyperpigmentation treatment with PL. However, due to the proven compound anti-inflammatory properties, the treatment of post-inflammatory hyperpigmentation could be possible with this compound [45].

## 4. Pycnogenol^®^

Pycnogenol^®^ is a product obtained from *Pinus pinaster* bark extract, containing phenolic acids (p-hydroxy benzoic, protocatechuic, gallic, vanillic, p-coumaric, caffeic and ferulic acids); condensed flavonoids (procyanidins); and monomeric phenolic compounds (catechin, epicatechin and taxifolin). Due to its composition, Pycnogenol^®^ is known for its anti-inflammatory and antioxidant activities [47,48,49,50,51].

Ni et al. [52] studied Pycnogenol^®^ for melasma treatment. Thirty women with melasma took a 25 mg tablet of Pycnogenol^®^ three times a day for 30 days. These volunteers were assessed clinically by melasma area index, pigmentary intensity index and by routine blood and urine tests. Efficacy was assessed at the 30th day of treatment, following the criteria: if a decrease in pigmentary intensity of melasma by two units or of the initial melasma area by more than one third was achieved and no new melasma appeared, the overall result was scored as 2; if the decrease in the pigmentary intensity was one unit and initial size was minor than one third, and no new melasma appeared, the overall result was scored as 1; when no change occurred in the pigmentary intensity, the result was scored as 0. The average pigmentary intensity of the volunteers decreased significantly on day 30, indicating an improvement of 0.47 units, and the average melasma area was also significantly reduced. Moreover, routine blood and urine test results were normal (blood cell count, hemoglobin and white blood cell count, urinalysis, among others).

Devaraj et al. [53] studied the effect of supplementation with 150 mg Pycnogenol^®^ for 6 weeks in the antioxidant capacity of the plasma and in its lipoprotein profile. During the study, the diet and routine activities of the volunteers were unchanged, but consumption of products rich in flavonoids was restricted. After a few weeks of supplementation with Pycnogenol^®^, blood samples of the volunteers were screened to evaluate the supplementation effects, and the same procedure was repeated when supplementation stopped. The results indicated an increase in plasma polyphenol levels during the supplementation period, demonstrating that the phenols from Pycnogenol^®^ were absorbed. On the other hand, a reverse effect on plasma polyphenol levels was observed when supplementation was interrupted. Other important findings were a significant decrease in LDL-cholesterol levels and an increase in HDL-cholesterol levels during supplementation. In contrast to LDL levels, HDL levels kept increasing after supplementation stopped, and this effect was observed in 66% of the volunteers. Even though the antioxidant effects of Pycnogenol^®^ were demonstrated in the study, the researchers pointed out that there was no evidence that these results could be entirely attributed to the antioxidant activity of Pycnogenol^®^.

Ryan et al. [54] studied the effect of Pycnogenol^®^ supplementation in an elderly population through cognitive and biochemical measures. The study showed that supplementation had a beneficial effect on the cognitive performance of the volunteers, more specifically an improvement in the working memory factor was observed. The working memory encompasses the capability of temporarily holding items from the spatial and numeric memories. However, no changes were determined on concentration and psychomotor abilities. In relation to the biochemical measures, a decrease in plasma F2-isoprostane concentrations suggested the biological effect of the antioxidant property of Pycnogenol^®^ to inhibit oxidative reactions.

Although these last investigations were not directly related to skin health, we decided to keep them in this review to highlight the Pycnogenol^®^ benefits in volunteers.

## 5. Carotenoids

Carotenoids are pigments present in plants, fungi, algae and several bacteria. In plants, for example, carotenoids play important roles in antioxidation, light absorption, providing color, and photoprotection [14,55,56].

The human body presents ways to protect itself against ROS using antioxidant system, such as endogenous antioxidants and enzymes. Exogenous antioxidants also play important role in this defense. Since carotenoids are potent antioxidants and humans do not synthesize them, supplementation is necessary when the intake of carotenoids in the diet is poor [55,57].

Several dietary supplements containing carotenoids are available on the market. For this review, we chose the carotenoids lutein and astaxanthin to discuss.

### 5.1. Astaxanthin

**Figure 2:**
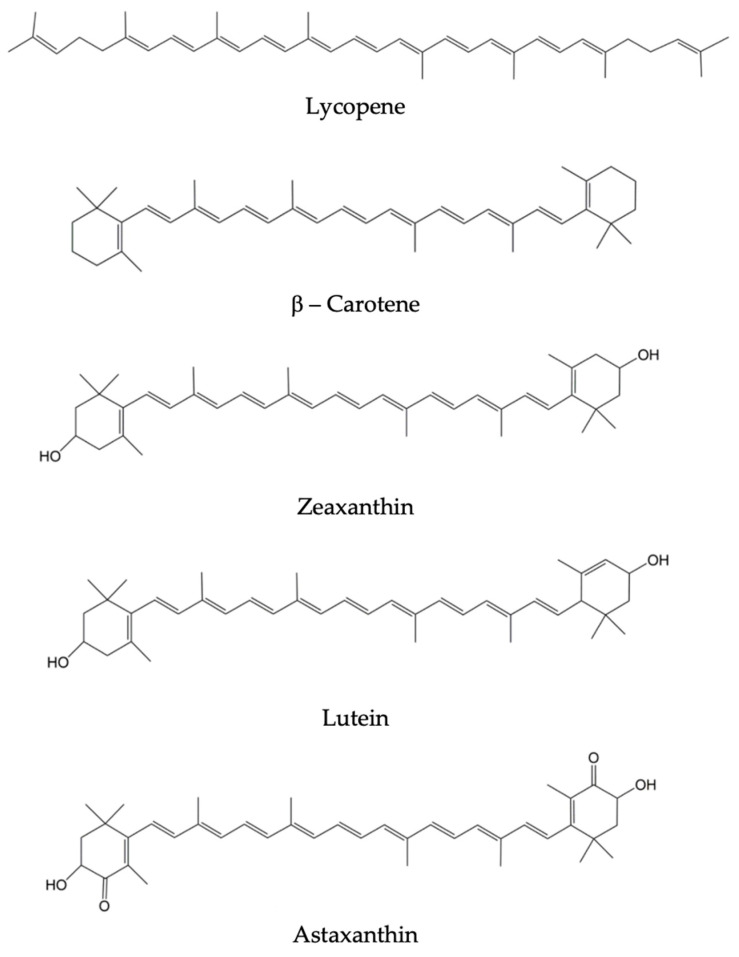
Molecular structures of selected carotenoids—adapted from [62].

Astaxanthin is a ketocarotenoid, 3,30-dihydroxy-b,b-carotene-4,40-dione, which was first isolated from lobsters by Kuhn and Sorensen in 1938 [58]. This red pigment is synthesized by plant, microorganisms and algae, and has several commercial applications, such as in food, nutraceuticals, pharmaceuticals, cosmetics and aquaculture industries. In aquaculture, for example, astaxanthin is used as a feed supplement to provide color to the salmon flesh [59]. Studies have demonstrated that astaxanthin performs many essential biological functions in marine species, such as providing pigmentation, protection against UV light damage and oxidation of macromolecules, and increase stress tolerance. Another important feature of astaxanthin is its antioxidant property, which is considered higher than that of β-carotene, zeaxanthin and lutein. Astaxanthin’s strong antioxidant capacity is attributed to the presence of keto and hydroxyl groups on its terminal ionone rings [60,61,62], as observed in Figure 2.

McNulty et al. [63] compared the activity of polar and apolar carotenoids (astaxanthin, β-carotene, lutein, lycopene and zeaxanthin) on the lipid peroxidation of membranes containing cholesterol. The results indicated a correlation between changes on the membrane structure caused by the addition of carotenoids and the extent of the lipid peroxidation. All carotenoids disturbed the membrane structure except astaxanthin, and these changes were attributed to the orientation and location of the carotenoids within the membrane. Furthermore, an increase in lipid peroxidation was noticed with the addition of β-carotene, lutein, lycopene and zeaxanthin, while astaxanthin led to its reduction. The researchers pointed out that the study was carried out with a membrane system less complex than biological membranes, but the findings still provide insights on the role of carotenoids on membrane lipid peroxidation.

Similarly, in a specialized review, Pashkow et al. [64] discussed the antioxidant property of astaxanthin in modulating oxidative stress. It was suggested in the study that a specific orientation of astaxanthin molecule in the cellular membranes allows its interactions with reactive species, which help to prevent membrane lipid peroxidation.

Another important feature of astaxanthin that has been extensively investigated is its cosmetic benefit to the skin. Tominaga et al. [65] evaluated the effect of astaxanthin administration to the skin of female and male volunteers. The female group received oral supplementation and topical treatment, while the male group only oral supplementation. Both groups showed wrinkle reduction and elasticity improvement after treatment. Additionally, the parameters age spot, skin texture and moisture content were assessed in the female group. A reduction in the age spot, an improvement in the skin texture and no significant difference in the moisture content were observed. Similar to the female group, the male group did not show any difference in the moisture content, but showed a significant improvement in the transepidermal water loss after treatment.

The same research group carried out in vitro and in vivo studies to determine the effects of astaxanthin administration on skin deterioration. Regarding the in vitro studies, the suppression of inflammatory cytokine secretion in keratinocytes and matrix metalloproteinase production by fibroblasts in samples treated with astaxanthin prior UVB irradiation was observed. Further studies, with volunteers, indicated a deterioration in the wrinkle’s parameters and moisture content in the placebo group; however, no significant changes were observed in the groups treated with a 6 mg and 12 mg dosage of astaxanthin. The level of inflammatory cytokine, IL-1α, increased in the placebo and low dosage groups, but not in the high dosage group. Moreover, an improvement in skin elasticity was observed only in the group treated with 12 mg of astaxanthin [58].

Another study on the effects of astaxanthin supplementation to the skin was developed by Chalyk et al. [3]. In the study, a group of middle-aged volunteers received astaxanthin supplementation, and their skin was examined before and after treatment. Changes on the components of the skin surface were evaluated, more specifically, changes on the morphology of the surface lipids, corneocyte desquamation and microbial presence. Additionally, the plasma level of malondialdehyde, a biomarker of the oxidative stress, was assessed to evaluate the antioxidant effect of astaxanthin. Regarding the surface lipids of the skin, their droplet size and crystal structure were evaluated. A slightly increase in droplet size was observed, whereas no clear changes on the crystal structure could be detected. Changes on the skin surface were indicated by a decrease in corneocyte desquamation and microbial presence. During astaxanthin supplementation, a steadily decrease in malondialdehyde concentration in the plasma was observed.

### 5.2. Lutein

Lutein and zeaxanthin belong to the group of carotenoids, lipophilic molecules that preferentially accumulate in the luteal macula, while other carotenoids, such as beta-carotene and lycopene, accumulate in the skin [5,57]. The molecular structures of lutein, zeaxanthin and lycopene are shown in Figure 2, and it is possible to observe hydroxyl groups on theirs terminal ionone rings, as a similarity [62].

A study developed by Palombo et al. [5] investigated the beneficial effects of lutein and zeaxanthin supplementation to the skin. Clinical trials employed topical, oral or combined (topical and oral) routes to administer the carotenoids. The study evaluated the supplementation effects on superficial skin lipids, hydration, lipid peroxidation, photoprotective activity and skin elasticity. All routes indicated a positive effect of lutein and zeaxanthin supplementation on the investigated parameters. However, the combined route showed the highest effect upon all skin physiological parameters except the skin elasticity. Overall, the results indicated that lutein and zeaxanthin supplementation was beneficial to the skin.

Another clinical study investigated the efficacy of lutein and zeaxanthin oral supplementation in healthy adults. The assessment parameters were skin tone and photoprotective activity. Skin lightening and luminance were improved with lutein/zeaxanthin supplementation when compared to the placebo group. Similar results were observed for the mean minimal erythema dose and the individual typological angle, since these parameters increased. Hence, the supplementation showed to improve skin conditions [57].

A comparative study of oral supplementation with lutein and lycopene evaluated human skin protection against UV radiation. This work investigated the influence of UVA/B and UVA 1 radiation in gene expression (HO1, ICAM1, and MMP1). The volunteers were divided in four groups: two groups started the treatment by either taking lutein or lycopene and then switched to placebo, while the other two groups first received placebo followed by carotenoid supplementation. Furthermore, the carotenoids were assessed in the blood samples during the treatment. Before and at the end of the supplementation period, the skin was irradiated and, subsequently, biopsies were performed after 24 h for gene expression analysis. The results suggested that both actives were protective against health damage induced by solar radiation, although lutein proved to be less effective when supplemented in the second period of the treatment. In addition, carotenoid blood levels significantly increased during the supplementation and returned to background levels when placebo was administered [4].

## 6. Tranexamic Acid

**Figure 3:**
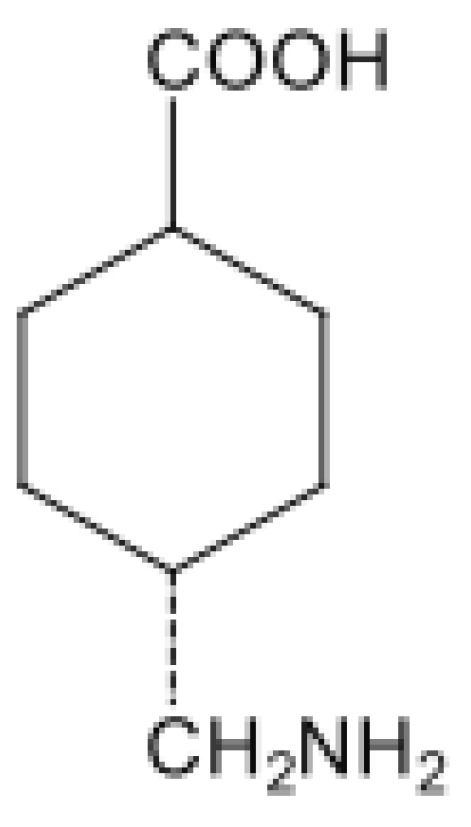
Molecular structure of tranexamic acid.

Tranexamic acid (Figure 3) is a synthetic derivative of the amino acid lysine and presents antifibrinolytic action. Its mechanism of action is due to its adhesion to the lysine binding sites, through a reversible blockade, in the plasminogen molecule, preventing it from binding to the activating factor of plasminogen. Once this blockade is created, plasmin (the main agent responsible for fibrinolysis) is no longer formed [66,67,68,69].

Another use of the tranexamic acid is on the treatment of melasma where its mechanism of action is associated with the blockage of the melanin synthesis. The effectiveness of the tranexamic acid in hyperpigmentation inhibition is attributed to its capacity to decrease tyrosinase activity and reverse melasma-related dermal changes [69,70,71,72].

Maeda and Naganuma [73] studied the effect of tranexamic acid on skin pigmentation induced by UV radiation in guinea pigs. The researchers topically applied solutions of tranexamic acid in different concentrations to the regions of animal skin already exposed to UV radiation. Histologically, by evaluating the amount of melanin in the control skin and treated with tranexamic acid, researchers found a suppression of melanin in the basal layer in the samples treated with the two more concentrated tranexamic acid solutions.

In a study developed by Karn et al. [74], the oral administration of tranexamic acid along with a routine treatment (topical hydroquinone and sunscreen) was evaluated on the treatment of melasma. The results indicated that the volunteers receiving the combined treatment showed a significant decrease in the mean melasma assessment severity index score, whereas those given the routine treatment had a significant decrease in the mean score only in the beginning, while the score remained stable in the last period of the treatment. Furthermore, the satisfaction assessment showed that 45.4% of the volunteers treated with the combined treatment graded it as excellent compared to only 8.5% of those who received the routine treatment.

Na et al. [75] conducted a clinical trial to investigate the effects of oral and topical administration of tranexamic acid on melasma treatment. Skin pigmentation and erythema were assessed to evaluate the treatment efficacy. Additionally, histological changes on the skin of selected volunteers were measured. The outcomes showed that the mean melanin index scores for the lesioned skin decreased during treatment, while the scores for the perilesional skin increased. The increase in the latter parameter was attributed to the period (spring to summer) that the study was carried out. Similar results were observed for the erythema index scores. Regarding the histological changes, the epidermal pigmentation showed a significant reduction, as well as a reduction in vessel numbers, vascularity and mast cell counts. Moreover, most of the volunteers observed an improvement on the skin appearance.

Another study, from Nagaraju et al. [76], also demonstrated the effects of tranexamic acid in skin with melasma. Thirty patients with refractory melasma received 500 mg of tranexamic acid orally, twice a day, associated with the use of a sunscreen. The assessment was based in the modified melasma area severity index (mMASI) and melasma quality of life index (MELASQOL) that were registered at baseline and after treatment. In addition, a skin biopsy was performed for immunohistochemistry and histopathology evaluation both before and after tranexamic acid supplementation. After a 4 month treatment, the results for mMASI assessment indicated that only one patient showed an excellent response. However, moderate and good improvement responses were perceived in 14 patients. In contrast, a slight improvement in the MELASQOL was observed in 4 patients, while a moderate improvement was seen in 23 patients and good improvement in 3. Concerning the histopathological evaluation, an epidermal pigmentation decrease was observed in 13 cases (representing 92.8% of the volunteers that went through skin biopsy). In addition, the inhibition of melanocyte proliferation was observed in 50% of the cases, and a reduction in melanin incontinence in 35,71% of the cases. Concerning the immunohistochemistry, among other interesting results, the researchers verified that Melan-A staining decreased with a statistical significance in all patients’ post-treatment.

## 7. Pomegranate (*Punica granatum* L.) Extract

*Punica granatum* L. extract is composed of several constituents, such as anthocyanins, polyphenols and tannins, for which multiple health benefits have been established. This plant is found in tropical and subtropical regions and is widely used for hypertension and atherosclerosis. A few in vitro and in vivo animal studies have already been carried out to evaluate the cutaneous impact of this extract, mainly in inhibiting melanin synthesis, improving hydration and elasticity and preventing skin ageing, in addition to antioxidant and anti-inflammatory activity and protection against damage from the UV exposure [1,77,78,79,80,81,82,83].

Kasai et al. [8] evaluated if the use of the pomegranate extract orally could protect the skin from UV radiation. In this study, volunteers aged from 20 to 40 years old were divided in three distinct groups with thirty volunteers each: placebo, those who received a higher dosage (200 mg) and those administered a lower dosage (100 mg) of the pomegranate extract. Before, during and after treatment (carried out for four weeks), the effect of UV irradiation on forearm skin was measured using a dose equivalent to 1.5 MED. Weekly measurements of erythema and melanin were established with the use of a Mexameter^®^. Even though no significant changes in the erythema and melanin values were observed, the volunteers reported an improvement on the skin appearance.

Henning et al. [77] evaluated in vivo the performance of oral supplementation of juice and capsules containing pomegranate extract against premature aging caused by UV radiation. This trial was carried out for 12 weeks on female volunteers aged between 30 to 40 years old and with skin phototype II to IV, which were separated into three distinct groups: placebo, those who consumed pomegranate juice and those who received supplementation with capsules containing 1000 mg of pomegranate extract. The study considered the equivalent number of polyphenols consumed by the two different supplements. The MED test and the skin melanin index (MI) were measured to ascertain the efficacy against the effects of skin radiation, both being determined before and after consumption of the active and/or placebo. Non-invasive equipment was used to measure MI, sebum on the skin surface, hydration and skin erythema before and after treatment. The UVB radiation dose and the exposure time were stipulated according to the skin type listed by the National Biological UVB mJ chart. The radiation was applied using a Dermalight^®^ 90 device in doses, which were in the range of 220–550 mJ/cm^2^ for a 100 to 290 s in previously defined areas in the arm. Twenty-four hours after irradiation, erythema formation was investigated through MED calculation. At the end of the study, it was found that treatment with pomegranate juice or extract provided a significant increase in the protection against erythema formation when compared to the placebo group, thus increasing MED. No significant changes in hydration and the amount of sebum in the skin were observed, and despite causing a non-significant reduction in melanin formation, the overall results seemed to indicate that the use of pomegranate extract can protect the skin from damage induced by UVB radiation.

## 8. Orthosilic Acid

Orthosilic acid (monomeric) is used to replace silicon in the human body since it has an efficient bioavailability when used orally. Silicon is present in the human body in small traces that, with ageing, begin to show a decrease in its levels; thus, its supplementation is recommended to provide skin benefits. Its supplementation has been associated with an improvement on the synthesis of collagen and elastin [9,84]. This compound is found in an accumulative form in the horny layer and in the hair fiber cuticle, since it can contribute to the improvement of the hair strands, stimulating growth and making them more resistant. In vivo studies have shown that the compound improves hair thickness and reduces the loss of elasticity of the capillary thread, promotes nail hardness, in addition to assisting in the skin barrier function and increasing the synthesis of collagen and elastin, preventing premature skin aging [9,84,85,86].

The use of orthosilic acid has long been the subject of in vivo studies to substantiate skin claims. In one of the most recent trials, it was observed that this component was able to promote a significant improvement in wrinkles and skin anisotropy in women aged 40 to 65 years old with signs of facial photoaging. These results were obtained by non-invasive bioengineering devices: viscoelastic properties and anisotropy by the Reviscometer^®^ MPA 5; skin anisotropy by the mechanical anisotropy indicator; and the presence of wrinkles was evaluated by the Visiometer^®^ SV 600. Measurements were conducted before and after oral supplementation for 20 weeks with capsules containing equivalent doses of 10 mg of silicon in the form of ch-OSA pellets (Bio Minerals n.v., Destelbergen, Belgium) twice daily [87].

In the research developed by Ferreira et al. [9], different forms of oral administration of elemental silicon were studied when stabilizing the structure with maltodextrin or monomethylsilanetriol. Volunteers, aged 40 to 60 years old with skin phototype III to V, were given a dose of 5 mg of silicon twice daily for 5 months and were divided in three groups: placebo, those that used a capsule containing maltodextrin and silicon, and those who were given an oral solution containing monomethylsilanetriol and silicon. Before and after the 3 and 5 month treatment, changes in the skin were evaluated using the Visia^®^ analysis system. At the end of the study, it was found that there was a significant improvement in facial wrinkles when comparing the groups that ingested the active with the placebo group. Both forms of administration promoted a statistically significant improvement of UV-induced pigmentation spots. Other parameters also showed significant differences among the groups, but these results were attributed to seasonal effects, since the study was carried out during different seasons.

Petersen et al. [85] aimed to evaluate the potential of stabilized silicon using a hydrolyzed marine collagen molecule (Exsynutriment^®^). This work was conducted on 22 male and female volunteers, between 40 and 60 years old, and involved 90 days of supplementation with 1 capsule a day. The volunteers were divided in two groups: those who received the active ingredient and those administered the placebo. Before the study, the volunteers were submitted to an evaluation by a dermatologist to determine the skin phototype according to the Fitzpatrick scale and the degree of skin aging according to the Glogau scale. In addition to these, the volunteers underwent a comparison of the characteristics of their face before and after the 90 days of treatment using images obtained by Visia^®^ (Canfield Imaging Systems Inc., Parsippany, NJ, USA), which allowed the evaluation of wrinkles, pores, damage caused by UV radiation and formation/alteration of skin spots. The skin characteristics of the groups (placebo and treated) showed no statistical differences at the start of treatment. However, after the treatment period, there was a significant difference in skin firmness (assessed by changes in the jowl, nasojulgal and eyelid folds and neck profile), texture (assessed by the presence of fine wrinkles and irregularities on the skin surface) and hydration (assessed by xerosis, scales, crust and shine); thus, it was concluded that this supplementation contributed to an improvement in skin conditions. However, orthosilic acid and hydrolyzed marine collagen were administered together; for this reason, it would be interesting to include a third group of volunteers who would receive only orthosilic acid, helping to understand which of the components, or if the combination of these, was responsible for the beneficial effect observed on the skin.

## 9. Conclusions

It is known that a healthy and balanced diet is of utmost importance for maintaining the health and functions of the body, including here the skin. Unfortunately, it is often not possible to eat so correctly; for this, food supplements become excellent allies. In this review, we discussed and presented studies on bioactive compounds that help to maintain the health and youthfulness of the skin. A wide variety of methodologies are available to ascertain the impact of food supplements in skin condition. For most of the substances or compounds herein, it was demonstrated that these products can act as adjuvants to obtain healthier skin, contributing to substantiate labelling claims, in addition to providing other significant benefits to the consumer. Overall, the following skin benefits from the consumption of these substances were proven: protection against oxidative stress, improvement in skin firmness, reduction in wrinkles and decrease in the pigmentary intensity of melasma, among others. We strongly believe that studies associating the oral use of food supplements with the application of cosmetic products containing the same bioactive compounds would elucidate and bring very interesting and even synergistic results, causing a positive impact on the skin.

## Data Availability

Not applicable.
